# The Associations of Perceived Self-Efficacy with Emotional Intelligence, Personality, Resilience, and Attitudes Towards Death among Midwives

**DOI:** 10.3390/healthcare12111129

**Published:** 2024-05-31

**Authors:** Evangelos Tzamakos, Dimitra Metallinou, Maria Tigka, Aikaterini Lykeridou, Antigoni Sarantaki, Christina Nanou

**Affiliations:** 1 Department of Midwifery, Faculty of Health and Care Sciences, University of West Attica, 12243 Athens, Greece; tzamvagpal@uniwa.gr (E.T.); klyker@uniwa.gr (A.L.); esarantaki@uniwa.gr (A.S.); nanouxv@uniwa.gr (C.N.); 2 Delivery Room, General and Maternity Hospital “Helena Venizelou”, 11521 Athens, Greece; maria.tigka@gmail.com

**Keywords:** perceived self-efficacy, emotional intelligence, personality, resilience, attitudes towards death, midwife, midwifery care, healthcare professionals

## Abstract

Midwives’ self-efficacy can significantly affect the provided care and, therefore, maternal and neonatal outcomes. The aim of the present study was to investigate associations of perceived self-efficacy with emotional intelligence, personality, resilience, and attitudes towards death among midwives in Greece. From 2020 to 2022, a total of 348 midwives were recruited in this descriptive cross-sectional study. The participants were employed as independent professionals, in public hospitals or regional health authorities. Data collection involved five research instruments: the General Self-Efficacy Scale (GSES), the Trait Emotional Intelligence Questionnaire-Short Form (TEIQue-SF), the Eysenck Personality Questionnaire (EPQ), the Connor-Davidson Resilience scale (CD-RISC), and the Death Attitude Profile-Revised (DAP-R) scale. The mean score for the GSES was 29.1 (SD = 4.2), suggesting a moderately elevated level of self-efficacy among midwives. The results revealed that higher scores on the GSES were significantly associated with higher scores on the Extraversion subscale (*p* < 0.001) and lower scores on the Neuroticism (*p* < 0.001) and Lie (*p* = 0.002) subscales of the EPQ. Additionally, high self-efficacy was significantly correlated with high emotional intelligence (*p* < 0.001), high neutral acceptance of death (*p* = 0.009), and high resilience (*p* < 0.001). These findings highlight the relationship between the self-efficacy of Greek midwives and various psychological factors, as well as the multifaceted nature of self-efficacy and its importance for midwives’ psychological well-being and professional functioning.

## 1. Introduction

Self-efficacy, a term coined by the psychologist Albert Bandura, refers to an individual’s belief in their capacity to execute behaviors necessary to produce specific performance attainments [1]. In the context of midwifery, perceived self-efficacy plays a crucial role in how midwives manage childbirth, face challenges, and support expecting mothers and their families through the prenatal, perinatal, and postnatal periods. The role of midwives is pivotal in ensuring safe and effective maternal and neonatal care, and often, these healthcare professionals become the primary caregivers for women and neonates during critical times [2,3,4]. Therefore, midwives’ confidence and belief in their abilities, termed as perceived self-efficacy, can significantly affect the care they provide and, consequently, the health outcomes of mothers and neonates/infants [5].

Principal factors influencing perceived self-efficacy in midwives include education and training, hands-on experience, mentorship, and the work environment. The depth and breadth of formal education and practical training can significantly enhance midwives’ confidence and competence in their work, while hands-on experience, especially in diverse and challenging situations, including maternal and neonatal critical care, tends to increase midwives’ self-efficacy by reinforcing their belief in their capabilities. For example, Maenhout et al. [6] explored the effect of repeated high-fidelity in situ simulation-based training on self-efficacy and reported that nursing and midwife self-efficacy in acute care situations was positively influenced by repeated simulation training in the NICU, regardless of the number of years of NICU experience. Moreover, access to mentorship and a supportive work environment can bolster a midwife’s confidence, providing her/him with resources and guidance when needed [7,8]. The conditions and culture within healthcare facilities and the work environment, including staffing levels, administrative support, and access to resources, can also have an impact on midwives’ perceived self-efficacy [9]. Night shifts, weekend shifts, lack of staff, and interprofessional continuing educational programs within the hospital environment, for instance, have been found to adversely influence self-efficacy [10,11].

The impact of perceived self-efficacy on midwives can be profound, influencing various aspects of their professional and personal lives. Midwives with high perceived self-efficacy are more likely to demonstrate competence and confidence in their abilities to deliver quick and effective care to mothers and neonates and make informed decisions and, therefore, lead to better decision-making, improved patient outcomes, and a higher quality of care overall [12]. As for stress and burnout, midwives who believe in their capacity to handle difficult situations are better equipped to manage stress, maintain their well-being, and prevent burnout. This resilience not only benefits their mental and emotional health but also ensures that they can continue to provide high-quality care [13].

High perceived self-efficacy has been also linked to greater job satisfaction, possibly because fewer feelings of frustration arise, and the professional is more able to overcome challenges and influence team dynamics and collaboration within healthcare settings [14,15]. Confident midwives are more likely to engage constructively with colleagues, advocate for their patients, and contribute positively to the team, leading to better coordinated and integrated care. Finally, the confidence that comes from high perceived self-efficacy can be communicated to patients, fostering a trusting relationship. When mothers perceive their midwives as proficient and self-assured, they are more likely to feel satisfied with their care, which can contribute to positive experiences [16].

Professional midwives’ self-efficacy has been previously examined in association with willingness to teach family planning [17] and comprehensive abortion care [18], the professional and working environment during the COVID-19 pandemic [19], their knowledge and skills based on the International Confederation of Midwives (ICM) competencies [12], labor-supportive behaviors [20], and postpartum hemorrhage management after interprofessional simulation training [21]. Attitudes towards death within the field of midwifery are multifaceted, and previous evidence suggests that these are influenced by sociodemographic factors, individual beliefs, professional experiences and training, and cultural features [22,23]. Midwives often regard death as a natural aspect of life, acknowledging their role in providing comfort and support during times of bereavement as well [24]. Emotional intelligence and personality traits, such as openness, cultivate professional resilience and acceptance, helping midwives manage emotionally demanding situations with empathy and inner poise and also ensuring their own well-being [25,26]. However, a limited body of evidence exists concerning midwives and the aforementioned psychometric tools, especially in our country. To the best of our knowledge, only a single Greek study [27] has incorporated midwives along with other healthcare professionals, all staff members of Neonatal Intensive Care Units, and examined them in relation to attitudes towards death. Regrettably, the limited number of participants (N = 131) made it impossible to conduct subgroup analysis across specialties, thus preventing the drawing of specific conclusions for midwives alone. No additional Greek studies have been identified that focus on the perceived self-efficacy, emotional intelligence, personality, and resilience of midwives.

As this study concentrates on the midwifery labor force, it is also crucial to include a brief analysis of the structure of midwifery services in Greece. Greek midwifery services are predominantly overseen by the Ministry of Health, which is responsible for setting the regulations, standards, and policies to guarantee both the safety and quality of midwifery care. Midwives are employed in various environments such as health centers, hospitals, family planning centers, assisted reproduction units, and home birth settings, tailored to the specific preferences and requirements of their patients. Midwifery education in Greece involves only direct-entry programs, which include a comprehensive 4-year university program culminating in a Bachelor’s degree [28]. This education generally adheres to the standards and directives of the European Union, ensuring consistency with European norms. Furthermore, assistant nurse midwives receive a 2-year basic training at Vocational Training Institutes, aimed at equipping them with the necessary skills and knowledge to support midwives and other healthcare professionals in maternity care settings. They serve a supporting role and do not operate independently. This role has been recognized in Greece since 2013, and to date, a relatively small number of assistant nurse midwives are actively employed [29].

In the context of the above evidence, we sought to investigate whether perceived self-efficacy correlated with emotional intelligence, personality, resilience, and attitudes towards death in Greek midwives.

## 2. Materials and Methods

### 2.1. Study Design and Ethical Considerations

This study took place between September 2020 and September 2022 using a descriptive cross-sectional design. Researchers recruited participants from four public tertiary hospitals, primary networks affiliated with two out of the seven Regional Health Authorities in Greece, and from direct email invitations targeting independent healthcare professionals within the authors’ professional network. Ethical approval for this study was obtained from the respective Committees of each participating hospital and Regional Health Authority involved in the study.

### 2.2. Sample and Setting

Eligible participants for this study were limited to midwives employed in the participating public hospitals, regional health authorities, or practicing independently within the prefecture of Attica, the Greek capital. For adherence to the inclusion criteria, midwives were required to (a) be proficient in Greek to facilitate completion of the study questionnaires and (b) hold a bachelor’s degree attained from either a technological educational institute or university, with a duration of study spanning 3.5 to 4 years. Assistant midwives who had completed a 2-year program were excluded from the study. Their exclusion helped to avoid confounding variables in our analysis and interpretation of the results, due to their distinct roles and responsibilities in the maternity settings, which derive from their different training.

A total of 500 midwives were invited to participate, of which 435 agreed to join in the study, yielding a response rate of 87%. However, final analysis of the data included only 348 professionals who completed all the requested instruments, five in total. Incomplete questionnaires or non-electronic submissions led to the exclusion of 87 midwives. Based on the 3504 active members registered with the Athens Midwives Association at the beginning of this study in 2020, the sample size of the study aligns with the established “10% rule”. Participants were recruited by random selection from the Athens Midwives Association register, taking into account that Athens, being the capital city of Greece, accounts for approximately 60% of the country’s midwives. The remaining 40% is distributed among six other Midwifery Associations in various prefectures of Greece.

### 2.3. Data Collection

This study adhered to the principles of quantitative methodology, using questionnaires as the main research instruments. Prior to participation, midwives were informed about the research objectives through a form, and their consent was obtained in accordance with the guidelines outlined in the General Data Protection Regulation (GDPR). 

Participants were given the option to complete the questionnaires either in print or online, particularly during periods of COVID-19 restrictions when in-person distribution was not feasible. We collected online data using a secure platform (Microsoft Forms), having been previously approved by the Ethics Committee of the university. Data quality assessments, authenticated responses, and response limits are some of the features and techniques that enhance the validity of responses on this platform. Participants were asked to submit the completed questionnaires within a week.

A preliminary estimation indicated that participants spent roughly 40 to 75 min completing all five research instruments. However, reading speed, comprehension, and decision-making ability can all affect the total amount of time required to fill in the questionnaires.

### 2.4. Research Instruments

In this study, midwives’ self-efficacy levels were evaluated using the General Self-Efficacy Scale (GSES), a self-report instrument consisting of 10 items. The GSES is used to detect associations among emotions, optimism, and employment satisfaction. Notably, negative coefficients are associated with depression, stress, health complaints, burnout, and anxiety. Responses are recorded on a 4-point Likert-type scale ranging from 1 (Not at all true) to 4 (Exactly true), and the total score is obtained by summing all items. Scores on the GSES range from 10 to 40, with higher scores indicating greater self-efficacy [30].

Furthermore, midwives’ attitudes towards death were evaluated utilizing the Death Attitude Profile-Revised (DAP-R) [31], which consists of five distinct subscales: fear of death, escape acceptance, approach acceptance, death avoidance, and neutral acceptance [32]. This self-report questionnaire comprises 32 items, employing a 7-point Likert-type scale ranging from 1 (strongly disagree) to 7 (strongly agree). Each subscale targets specific aspects of attitudes towards death, such as negative thoughts and feelings related to death (fear of death), considering death as a relief from suffering (escape acceptance), viewing death as an entrance to a better afterlife (approach acceptance), efforts to avoid thinking about death (death avoidance), and acceptance of death as a natural aspect of life (neutral acceptance).

Mental resilience was assessed using the Connor-Davidson Resilience Scale (CD-RISC), a 25-item self-report questionnaire [33]. Each question is scored on a scale from 0 to 4, and the total score reflects the individual’s overall level of mental resilience. Resilience is conceptualized as the ability to effectively cope with stress and adversity, making it a potentially valuable target for interventions addressing stress reactions, anxiety, and depression.

Additionally, participants’ emotional intelligence was measured through the Trait Emotional Intelligence Questionnaire-Short Form (TEIQue-SF) [34,35]. This questionnaire comprises 30 items, each rated on a scale from 1 to 7, with higher scores indicating greater emotional intelligence. While the TEIQue-SF is primarily designed to measure overall emotional intelligence, Petridis (2009) identified four distinct subscales: emotionality, self-control, sociability, and well-being [34].

The personality traits of the study cohort were evaluated using the Eysenck Personality Questionnaire (EPQ) [36]. The EPQ measures several subscales including Extraversion, Neuroticism, Psychoticism, and a Lie subscale. Comprising 84 closed-ended questions answered with yes/no responses, the EPQ provides insights into various personality dimensions. Extraversion reflects sociability and impulsivity, with individuals scoring high in this dimension typically energetic and enjoying social interactions. Neuroticism indicates emotional instability and reactivity, with high scorers often experiencing feelings of guilt, shyness, anxiety, and depression and possessing low self-esteem. Psychoticism reveals traits such as insensitivity, distance, irrationality, and a lack of empathy for others. Additionally, the Lie subscale was designed to assess tendencies towards dissimulation [37].

Each of the research instruments used in the present study, including the GSES, DAP-R, CD-RISC, TEIQue-SF, and EPQ, calculated reliability coefficients (Cronbach’s alpha) above 0.70, indicating satisfactory levels of internal consistency for each respective scale [38]. All research instruments have been translated and validated in the Greek population [31,35,39,40,41,42].

### 2.5. Statistical Analysis

Quantitative variables were described using both mean values (Standard Deviation) and median (interquartile range). Categorical variables were detailed using absolute and relative frequencies. Spearman correlation coefficients (ρ) were employed to examine the associations between pairs of continuous variables. Hierarchical linear regression was conducted to identify factors that may be independently associated with participants’ self-efficacy. In the first step of the analysis, all participants’ demographic and employment information were entered (method: enter). In subsequent steps, DAP-R, EPQ, TEIQue-SF and CD-RISC scores were entered using the stepwise method (*p* for entry 0.05, *p* for removal 0.10). Linear regression analyses results were utilized to compute adjusted regression coefficients (β) with standard errors (SEs) and standardized coefficients (beta). Hierarchical linear regression was conducted after log-transforming the GSES scale due to the non-normal normal distribution of the data. Internal consistency reliability was assessed by calculating Cronbach’s alpha coefficient, with scales deemed acceptable if they had a reliability of 0.70 or higher. All reported *p* values are two-tailed. Statistical significance was established at *p* < 0.05. Analyses were conducted using SPSS statistical software (version 26.0).

## 3. Results

The study dataset comprised information from 348 midwives. Most participants were female (92.8%), aged between 41 and 50 years (33.9%), held a Master of Science (MSc) degree (49.1%), and were married (68.4%). Furthermore, 71.6% of the participants were parents, with 46.8% reporting having two children. On average, participants had accumulated 17.1 years of professional experience (SD = 9.5 years), with an average tenure of 9.9 years in their current department (SD = 7.8 years). Detailed characteristics of the participants are outlined in Table 1.

Table 2 presents the descriptive statistics of all scales under study, along with their respective reliability coefficients. The mean score for the GSES was 29.1 (SD = 4.2).

Higher scores on the GSES were found to be significantly associated with higher scores on the Extraversion subscale and lower scores on the Neuroticism and Lie subscales (Table 3). Additionally, higher GSES scores were significantly associated with higher scores on the TEIQue-SF, the neutral acceptance subscale of DAP-R scale, and the CD-RISC.

The results of the hierarchical linear regression analysis, with the GSES as the dependent variable, are summarized in Table 4. The analysis revealed a significant positive association between higher levels of emotional intelligence, as measured by the TEIQue-SF score, and greater self-efficacy. Participants’ demographic and employment characteristics, however, did not significantly correlate with self-efficacy levels in this study.

## 4. Discussion

For the first time, perceived self-efficacy among certified midwives was examined whether it correlates with various psychological constructs, including emotional intelligence, personality traits, resilience, and attitudes towards death. By integrating five distinct research tools in one study—GSES, DAP-R, CD-RISC, TEIQue-SF, and EPQ—a significant contribution has been made to the understanding of these relationships, particularly in the context of the Greek midwifery workforce.

In the present study, midwives reported a mean score for the GSES of 29.1 (SD = 4.2). This finding indicates that, on average, midwives in the studied population reported a moderately elevated level of self-efficacy as reflected in their responses to the scale’s items. The results suggest that participants harbor a confident belief in their ability to effectively cope with a variety of challenging situations and emergencies when providing midwifery care. A recent Polish study demonstrated a particularly high sense of self-efficacy among midwives employed in the delivery room regarding the use of non-pharmacological methods of relieving labor pain, with a mean score of 30 points (SD = 3.3) on the GSES [43]. On the contrary, the mean score of self-efficacy among midwives in a multi-center Chinese study was quite low, reaching a score of 24.34 (SD = 5.28) on the GSES, which was linked to low personal accomplishment, emotional exhaustion, and job burnout [13]. Burnout is characterized by negative attitudes towards work, which can lead to a decrease in interest and reduced professional performance [44]. Notably, as far as midwives are concerned, it has been reported that their strong belief in their ability to cope with challenging situations positively influences the promotion of natural births [45]. Midwives perceive that confidence in their abilities correlates with their competence and professional performance, emphasizing the importance of self-efficacy in midwifery practice [8].

The findings of the present study revealed that higher scores on the GSES were significantly associated with higher scores on the Extraversion subscale and lower scores on the Neuroticism and Lie subscales of the EPQ. Firstly, the combination of self-efficacy and Extraversion could suggest that midwives with such traits are not only confident in their abilities but also socially active and assertive. These two qualities are often found in effective leaders, and midwifery leadership needs guidance, inspiration, and influence on colleagues, including both other midwives and the wider multidisciplinary team [46]. Additionally, individuals with high self-efficacy and Extraversion are likely to set high goals and make significant achievements, including academic ones [47], and this association could possibly be explained by the fact that a high percentage of our studied sample had received an MSc and a PhD degree. Secondly, when high self-efficacy is paired with low Neuroticism, it suggests a dynamic personality with emotional stability, optimism, and resilience, and it has been found that such traits can predict academic performance and attrition in nursing students and potentially assist with psychological profiling which might lead to a more effective selection process [48]. Finally, when high self-efficacy is correlated with low scores on the Lie subscale, it indicates a personality profile characterized by confidence in one’s abilities, greater honesty, straightforwardness, and authenticity. The provision of high-quality midwifery care requires the promotion of trusting relationships and the integration of decision-making during pregnancy and childbirth, as it is of utmost importance for women to have positive birth experiences [49,50]. 

Furthermore, in the context of midwifery, the correlation of high self-efficacy with high emotional intelligence seems critical as it might contribute significantly to the effectiveness and satisfaction levels of midwives in their professional roles. An unpublished study focused on midwives in Northern Iran revealed that their job satisfaction and emotional intelligence were moderate on average [51], and it also highlighted that higher levels of emotional intelligence were associated with increased job satisfaction among midwives. This correlation included improved inter- and intrapersonal skills and adaptability, suggesting that as midwives’ abilities to understand and manage their own emotions and adapt to changing circumstances improve, their satisfaction with their job also increases. Finally, this correlation was remarkable in the broader context of team performance and cohesion, aspects undoubtedly essential to the midwifery profession [52]. In our study, midwives’ mean time of total professional experience was 17.1 years and 9.9 years in the department they worked in at the time the study was conducted, which are findings that indicate our studied sample was satisfied with the midwifery profession overall.

Additionally, the association between higher scores on the GSES and higher scores on the neutral acceptance subscale of the DAP-R indicates that midwives with greater general self-efficacy tend to accept death as a natural aspect of life, without significant fear or welcome. However, accepting mortality and viewing death as an inevitable part of the human experience rather than something to be feared or avoided excessively can be emotionally difficult, especially for midwives who work in settings where maternal, perinatal, and neonatal deaths occur, resulting even in occupational trauma [53]. In a previous Greek study as well, conducted among healthcare professionals working in neonatal intensive care units [27], including midwives in the highest percentage, it was found that greater ‘frequency of care related to end of life neonates’ was significantly associated with greater fear of death, while neutral acceptance was not associated significantly with any of the studied characteristics and experiences.

Finally, higher GSES scores were significantly associated with higher scores on the CD-RISC, indicating that midwives with higher self-efficacy also tend to exhibit greater resilience in coping with the demanding and stressful nature of their profession. The COVID-19 crisis, for instance, was a challenging period for midwives, as it disrupted and intensified pre-existing stressors and adversities which further affected midwives’ ability to practice within their professional norms [54]. A recent systematic review underscored the importance of resilience as a process revealed over time through person–environment interactions, which can be positively influenced by internal resources like self-efficacy and external resources such as supportive professional relationships and a safe work environment. Programs aimed at strengthening these factors have shown promising results in reducing stress and burnout symptoms while increasing self-efficacy and resilience [55].

The results of the hierarchical linear regression analysis showed that participants’ demographic (sex, age, educational level, marital status) and employment characteristics (years of experience) did not significantly correlate with self-efficacy levels in this study. On the contrary, Jiang et al. [13] reported significant differences in the self-efficacy of midwives of different age, marital status, and length of service but none as far as it concerns the educational level. 

The present study is subject to inherent limitations that require careful consideration when interpreting its findings. Despite the use of standardized research instruments known for their sensitivity in capturing different traits and characteristics, all responses focused on the subjective perceptions of participants rather than objective criteria. Reliance on self-report measures introduces the possibility of response bias and social desirability effects, where participants may provide answers, they perceive as favorable rather than reflecting their actual beliefs or behaviors. Consequently, there is the possibility of false positives. In our effort to reduce selection bias, we tried to select participants from different healthcare settings and by random sampling. The studied sample was recruited from only one city, the capital of Greece, and may not fully represent the entire Greek midwifery workforce, ultimately limiting the generalizability of the findings. Additionally, the extensive time required to complete the research instruments could indeed be a factor influencing participants’ decision to participate in the study, leading some to return incomplete questionnaires. This could have resulted in a biased sample, potentially affecting the representativeness of the study population and the generalizability of the results. Finally, this study did not designate variables as confounding factors; therefore, no specific strategies were used to manage and minimize confounding bias in the present study. 

Future research could delve deeper into developing interventions aimed at enhancing midwives’ self-efficacy, eventually incorporating training programs or supportive mentorship initiatives. Exploring the influence of specific job demands and stressors on midwives’ self-efficacy levels could offer targeted strategies to mitigate workplace challenges and enhance overall well-being. Additionally, exploring the role of organizational support and workplace culture in fostering self-efficacy among midwives could provide valuable insights for creating supportive environments that promote professional growth and resilience. Longitudinal studies could be conducted to explore the dynamic nature of self-efficacy among midwives over time and its impact on job satisfaction, burnout rates, and patient care outcomes. Further examination of the interplay between self-efficacy, coping mechanisms, and job-related outcomes could help to elucidate the mechanisms through which self-efficacy influences midwifery practice and patient care.

## 5. Conclusions

This study highlights the relationship between the self-efficacy of Greek midwives and various psychological factors. The findings indicate that midwives generally demonstrate moderately elevated levels of self-efficacy and believe in their ability to cope with challenging situations and emergencies when providing midwifery care. Self-efficacy is associated with personality traits, emotional intelligence, and attitudes towards death and resilience. Interestingly, demographic factors do not significantly influence levels of self-efficacy. These results highlight the multifaceted nature of self-efficacy and its importance for midwives’ psychological well-being and professional functioning. Further research on interventions aimed at enhancing self-efficacy among midwives could yield valuable insights into improving professional satisfaction and patient outcomes in midwifery practice.

## Figures and Tables

**Table 1:** Participants’ demographic and socioeconomic characteristics (N = 348; Attica, Greece, 2020–2022).

Variables		N (%)
Sex	Male	25 (7.2)
	Female	323 (92.8)
Age (years)	20–30	48 (13.8)
	31–40	96 (27.6)
	41–50	118 (33.9)
	≥51	86 (24.7)
Educational level	Tertiary education	162 (46.6)
	MSc	171 (49.1)
	PhD	15 (4.3)
Family status	Unmarried	78 (22.4)
	Married	238 (68.4)
	Divorced	25 (7.2)
	Widower	7 (2.0)
Number of children	0	99 (28.4)
	1	48 (13.8)
	2	163 (46.8)
	3	37 (10.6)
	4	1 (0.3)
Years of professional experience, Mean (SD)		17.1 (9.5)
Professional experience in the present department, Mean (SD)		9.9 (7.8)

**Table 2:** Descriptive statistics and reliability coefficients for General Self Efficacy Scale (GSES), Trait Emotional Intelligence Questionnaire-Short Form (TEIQue-SF), Eysenck Personality Questionnaire (EPQ), Connor-Davidson Resilience Scale (CD-RISC), and Death Attitude Profile-Revised (DAP-R) scale. (N = 348; Attica, Greece, 2020–2022).

Research Instruments	Measurements				
	Minimum	Maximum	Mean (SD)	Median (IQR)	Cronbach’s a
GSES	15.0	40.0	29.1(4.2)	29.0 (27–31)	0.87
TEIQue-SF	2.5	6.5	5 (0.6)	5.1 (4.6–5.4)	0.82
EPQ					
Psychoticism	0.0	13.0	4 (2.3)	4 (2–5)	0.71
Extraversion	4.0	19.0	14 (3.6)	14.5 (12–17)	0.77
Neuroticism	1.0	21.0	10.8 (4.4)	11 (7–14)	0.80
Lie	1.0	16.0	7.6 (3.2)	8 (5–10)	0.72
CD-RISC	25.0	98.0	68.6 (12.5)	29.0 (27–31)	0.91
DAP-R					
Death avoidance	1.00	7.00	3.5 (1.4)	3.4 (2.2–4.6)	0.83
Neutral acceptance	1.00	6.80	2.9 (0.8)	2.8 (2.4–3.4)	0.71
Approach acceptance	1.00	7.00	3.9 (1.2)	3.8 (3.2–4.6)	0.76
Fear of death	1.00	6.57	3.3 (1.2)	3.1 (2.6–4.0)	0.80
Escape acceptance	1.40	6.70	4.6 (1.0)	4.7 (3.9–5.3)	0.77

**Table 3:** Correlation analysis of General Self-Efficacy Scale (GSES) with Trait Emotional Intelligence Questionnaire-Short Form (TEIQue-SF), Connor-Davidson Resilience Scale (CD-RISC), Eysenck Personality Questionnaire (EPQ), and Death Attitude Profile-Revised (DAP-R) scale. (N = 348; Attica, Greece, 2020–2022).

Spearman Correlations between Variables		
		General Self-Efficacy (GSES) scale
TEIQue-SF	ρ ^1^	0.56
	*p* ^2^	<0.001
CD-RISC	ρ	0.67
	*p*	<0.001
EPQ		
Psychoticism	ρ	0.08
	*p*	0.154
Extraversion	ρ	0.36
	*p*	<0.001
Neuroticism	ρ	−0.31
	*p*	<0.001
Lie	ρ	−0.17
	*p*	0.002
DAP-R		
Death avoidance	ρ	0.02
	*p*	0.664
Neutral acceptance	ρ	0.14
	*p*	0.009
Approach acceptance	ρ	−0.09
	*p*	0.103
Fear of death	ρ	0.02
	*p*	0.698
Escape acceptance	ρ	0.03
	*p*	0.529

^1^ ρ: for Spearman correlation coefficient. ^2^ *p*: for *p*-value.

**Table 4:** Hierarchical linear regression analysis results with General Self-Efficacy (GSES) scale as the dependent variable. (N = 348; Attica, Greece, 2020–2022).

Independent Variables ^1^	β +	SE ++	b ‡	*p*
Sex (Female vs. Male)	−0.014	0.011	−0.054	0.222
Age				
31–40 years vs. 20–30 years	0.008	0.010	0.057	0.453
41–50 years vs. 20–30 years	−0.003	0.014	−0.021	0.836
≥51 years vs. 20–30 years	−0.025	0.018	−0.164	0.178
Educational level (MSc/PhD vs. Tertiary education)	−0.005	0.006	−0.038	0.419
Family status: Married (Yes vs. No)	−0.003	0.007	−0.059	0.501
Years of professional experience	0.001	0.001	0.158	0.081
TEIQue-SF	0.030	0.006	0.291	<0.001

^1^ The results presented are the final step of the hierarchical linear regression. + regression coefficient, ++ Standard error, ‡ standardized coefficient.

## Data Availability

Data are contained within the article.
